# Semi-Automated Extraction of the Distribution of Single Defects for nMOS Transistors

**DOI:** 10.3390/mi11040446

**Published:** 2020-04-23

**Authors:** Bernhard Stampfer, Franz Schanovsky, Tibor Grasser, Michael Waltl

**Affiliations:** 1 Institute for Microelectronics, Technische Universität Wien, Gusshausstraße 27-29/E360, 1040 Wien, Austria; grasser@iue.tuwien.ac.at (T.G.); waltl@iue.tuwien.ac.at (M.W.); 2 Global TCAD Solutions, Bösendorferstraße 1/12, 1010 Wien, Austria; f.schanovsky@globaltcad.com

**Keywords:** MOSFET, reliability, random telegraph noise, oxide defects, SiO_2_

## Abstract

Miniaturization of metal-oxide-semiconductor field effect transistors (MOSFETs) is typically beneficial for their operating characteristics, such as switching speed and power consumption, but at the same time miniaturization also leads to increased variability among nominally identical devices. Adverse effects due to oxide traps in particular become a serious issue for device performance and reliability. While the average number of defects per device is lower for scaled devices, the impact of the oxide defects is significantly more pronounced than in large area transistors. This combination enables the investigation of charge transitions of single defects. In this study, we perform random telegraph noise (RTN) measurements on about 300 devices to statistically characterize oxide defects in a Si/SiO${}_{2}$ technology. To extract the noise parameters from the measurements, we make use of the Canny edge detector. From the data, we obtain distributions of the step heights of defects, i.e., their impact on the threshold voltage of the devices. Detailed measurements of a subset of the defects further allow us to extract their vertical position in the oxide and their trap level using both analytical estimations and full numerical simulations. Contrary to published literature data, we observe a bimodal distribution of step heights, while the extracted distribution of trap levels agrees well with recent studies.

## 1. Introduction

The amorphous SiO${}_{2}$ used as the insulator material in silicon metal-oxide-semiconductor field effect transistors (MOSFETs) contains electrically active defects, often referred to as traps. These traps cause a number of effects detrimental to the operating characteristics and reliability of the devices [1,2]. The most prominent reliability issues in these devices related to charge trapping are the so called bias temperature instabilities (BTI) [3,4,5] and random telegraph noise (RTN) [6,7,8]. While BTI can be observed in large-area and nanoscale devices, RTN is mainly studied on scaled technologies.

**Figure 1:**
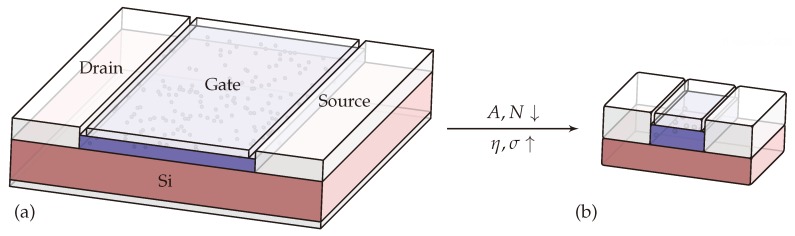
Illustration of oxide defects in (**a**) large- and (**b**) small area devices. As the gate area *A* is reduced, fewer defects *N* will be present in the device, but on average each defect will have a larger impact η on the overall degradation. This increases variability σ among nominally identical devices, but also allows characterization of single defects using methods such as random telegraph noise (RTN) analysis and time-dependent defect spectroscopy (TDDS).

With shrinking of the gate area of a MOSFET, the average number of defects present in the oxide decreases, see Figure 1. The smaller number of defects, however, does not lead to a reduction in threshold voltage shift or noise power, as one might intuitively expect. This is due to the larger influence defects in scaled devices have on the channel [9,10]. Below a certain gate area, single charge transitions can be observed as discrete steps in the drain current, enabling the characterization of RTN. While for large-area and nanoscale devices a similar average degradation of the threshold voltage can be observed, device-to-device variability is seriously affected by device scaling [11,12]. As a consequence, accurate characterization of nanoscale devices requires a higher number of samples than required for large area device studies. For this, we measure RTN on more than 300 scaled devices to evaluate the impact of defects on these devices. We further analyze part of the defects in more detail using an analytical approximation, to determine their distribution in depth and energy. Finally, we use numerical defect simulation to reproduce some of the defects’ charge trapping kinetics and compare the obtained distributions to the distributions from the analytical approximation and literature data.

## 2. Materials and Methods

In the following, the devices used in this work and the measurements carried out are discussed. Afterwards, an efficient method to analyze the recorded data is given. Finally, an analytical approximation for the trap level and the trap position is discussed. Using the proposed methodology the trap bands can be extracted from the measurement data. While the focus of this study is on Si/SiO${}_{2}$ devices, the methodology is possible on other technologies, given that small devices are available which are also stable enough to record RTN in a reproducible fashion [13,14,15].

### 2.1. Devices and Measurements

The devices investigated in this study are planar Si/SiO${}_{2}$ devices with an effective gate area of A≈ $0.15\;\mu m^{2}$. For all measurements, we use a custom defect probing instrument (DPI) [16] which is configured with three voltage sources for the gate, drain, and bulk terminals of the transistor and a low-noise trans-impedance amplifier with switchable amplification, in combination with a sampling input for measuring drain-source current $I_{\mathrm{DS}}$, connected to the source of the device. The devices are contacted using a Cascade/FormFactor PA300 semi-automatic wafer prober (FormFactor, Inc., Livermore, CA, USA), which is fully shielded. Electrical connections between the prober and the measurement instrument are made with double shielded TRIAX cables (Keithley Instruments, Solon, OH, USA) to further reduce spurious noise.

**Figure 2:**
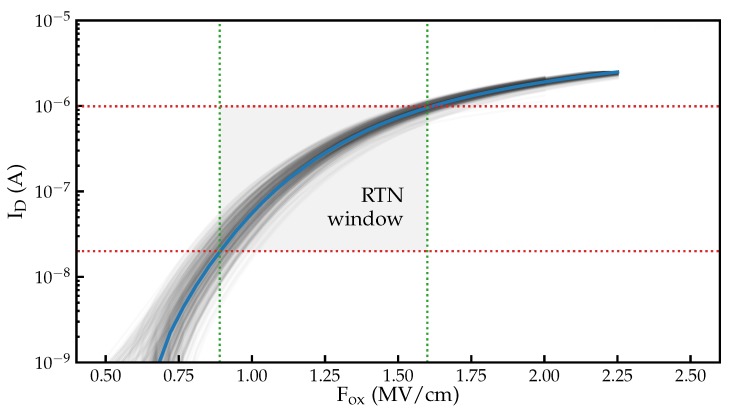
Transfer characteristics measured on approximately 300 nominally identical devices, with the average characteristic indicated in blue. The bias range (green) and current range (red) used for RTN measurements in this work is indicated using dotted lines.

To obtain statistics about the defects active in the devices, automated measurements are performed on around 300 devices. In our measurement scheme, the transfer characteristic is recorded for each device, followed by RTN measurements. The initial $I_{D}$($V_{G}$) curve is used to verify proper operation of the tested device, to determine at which voltages the RTN signals are measured, and to map the recorded $I_{D}$(t) RTN data to $\Delta V_{\mathrm{th}}$(t). In order to minimize stress to the device prior the RTN measurement, the initial $I_{D}$($V_{G}$) characteristics are recorded within a narrow gate bias window; see Figure 2.

The RTN traces are recorded at the gate voltages corresponding to 20, 50, 100, 300, and 1000 nA for each device. These values are selected to achieve maximum current resolution for the measurements ranges available. The drain-source voltage used for both the $I_{D}$($V_{G}$)and RTN measurements is −100 mV.

**Figure 3:**
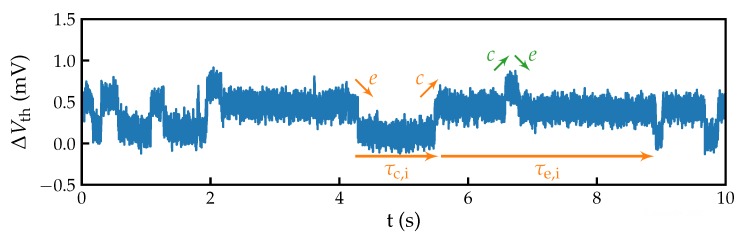
Random telegraph noise traces recorded on a device with $T_{s}=100\;\mu s$. Two active defects with similar step heights are clearly visible. The arrows show steps and dwelling times linked to charge capture (c) and charge emission (e) events of the defects.

For each gate bias point, one long and five short RTN traces are recorded. The long traces feature a sampling time of $T_{s}=10\;ms$ and a total recording time of $t_{r}=1\;ks$, while the short traces are sampled with $T_{s}=100\;\mu s$ for $t_{r}=10\;s$. This allows us to characterize defects with a wider range of transition times, while keeping the number of samples per trace manageable [17]. An example of a fast-sampled RTN trace recorded is shown in Figure 3. The trace was mapped from $I_{D}$(t) to $\Delta V_{\mathrm{th}}$(t). For this, each $I_{D}$ value in the trace is replaced with the corresponding $V_{G}$ from the initial $I_{D}$($V_{G}$), and finally subtracted from the applied $V_{G}$ [18].

To characterize individual defects in detail, additional RTN measurements are performed on selected devices, with measurement parameters tailored to the defects under investigation. All measurements are performed at 30 ${}^{\circ }$C unless indicated otherwise.

### 2.2. Noise Parameter Extraction

To obtain the average step heights η as well as the charge capture and charge emission times $\tau_{c},e$ of the defect signals comprising the recorded traces, a 1D variant of the Canny edge detector [19,20] is first applied on the $\Delta V_{\mathrm{th}}$ data. This method is chosen because (i) there are only few defects per device on average, (ii) the signal-to-noise ratio is reasonably high, and (iii) the method requires little manual interaction. Other methods which could be used for this step include time-lag methods [21,22] or methods based on hidden Markov models [23,24].

To determine the positions of the steps, the $\Delta V_{\mathrm{th}}$(t) data is convoluted with the first derivative of a Gaussian pulse with a chosen standard deviation $\sigma_{g}$, truncated in time to $\pm L=5\sigma_{g}$:

(1) $$h\left(t\right)=\Delta V_{\mathrm{th}}\left(t\right)*g\left(t\right)=\int_{-L}^{L}\Delta V_{\mathrm{th}}\left(t-\delta \right)g\left(\delta \right)d\delta$$

**Figure 4:**
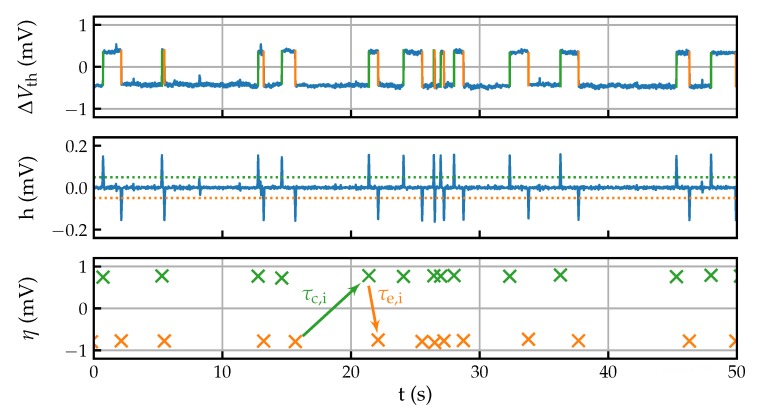
Noise parameter extraction using the Canny algorithm. To find steps in a $\Delta V_{\mathrm{th}}$ trace, it is convoluted with the first derivative of a Gaussian pulse. This yields a signal *h*, as shown in the center panel, with peaks at the positions of the steps. Thresholding is then applied to *h* to suppress noise. Finally, the positions of the steps are obtained from the positions of the local maxima in *h*. The height of the steps may be obtained either from the peaks in *h* or from the original trace. The result is a list of steps ($t_{i}$, $\eta_{i}$) from which, ideally, the average step height η, capture time $\tau_{c}$, and emission time $\tau_{e}$ for each defect can be extracted.

The resulting signal is then compared to a threshold value, which is chosen to suppress spurious responses. Local maxima in the signal above this threshold level then give the locations of steps $t_{i}$ in the original trace. With the positions of the steps, their height $\eta_{i}$ can be determined from the original signal, resulting in a list of steps ($t_{i},\eta_{i}$) as shown in Figure 4. From the list of steps, the average step heights and transition times of the defect responses can be determined, given the defects are distinguishable in step height. If this is not the case, i.e., multiple defects with similar step heights contribute to a trace, this may lead to erroneous results. This is indicated in the data by successive positive or negative steps and a non-exponential distribution of the calculated transition times.

### 2.3. Defect Parameter Estimation

From defects which are close to the Fermi level at measurement conditions, spatial and energetic positions can be estimated from their capture and emission times measured at a number of voltages [6,25,26]. The central assumption for this estimation is that a defect at the Fermi level $E_{t}=E_{f}$ has an occupancy of 50%, i.e., $\tau_{c}=\tau_{e}$. It is further assumed that the rate equations for charge capture from the channel, and charge emission to the channel can be written in the form:

(2) $$k_{c},e=k_{0}c,e\exp\left(-\frac{E_{c},e}{k_{B}T}\right),$$

with the capture and emission rates $k_{c},e=1/\tau_{c},e$, prefactors $k_{0}c,e$, energy barriers for capture and emission $E_{c},e$, the Boltzmann constant $k_{B}$, and the device temperature *T*. From the logarithm of 2:

(3) $$\ln k_{c},e=\ln k_{0}c,e-\frac{E_{c},e}{k_{B}T}$$

its dependence on the gate bias is expressed:

(4) $$\frac{\partial \ln k_{c},e}{\partial V_{G}}=\frac{\partial \ln k_{0}c,e}{\partial V_{G}}-\frac{1}{k_{B}T}\frac{\partial E_{c},e}{\partial V_{G}}.$$

Assuming the bias dependence of the prefactor can be neglected, and subtracting 4 for capture and emission yields

(5) $$\frac{\partial \ln k_{c}/k_{e}}{\partial V_{G}}=-\frac{1}{k_{B}T}\frac{\partial (E_{c}-E_{e})}{\partial V_{G}}=-\frac{1}{k_{B}T}\frac{\partial E_{t}}{\partial V_{G}}.$$

Here, $\partial (E_{c}-E_{e})$ is replaced by the change in trap level $\partial E_{t}$. This is possible due to

(6) $$E_{c}-E_{e}=\left(E_{\max}-E_{r}\right)-\left(E_{\max}-E_{t}\right)=E_{t}-E_{r}$$

where the bias independent reservoir energy $E_{r}$ and the peak of the energetic barrier $E_{\max}$ [27]. Note that this makes no assumptions on the shape of the energetic barriers between the two states of the system. Assuming the device is operating in inversion and a homogeneous oxide field is applied, the change in effective trap energy with gate voltage can be expressed with the position of the defect in the oxide as

(7) $$\frac{\partial E_{t}}{\partial V_{G}}=-q\frac{d}{t_{\mathrm{ox}}}.$$

Equations (5) and (7) yield the relative position of the defect in the oxide

(8) $$\frac{d}{t_{\mathrm{ox}}}=-\frac{k_{B}T}{q}\left(\frac{\partial \ln\tau_{c}/\tau_{e}}{\partial V_{G}}\right),$$

written with $\tau_{c},e=1/k_{c},e$. Integrating Equation (7) gives the thermodynamic trap level of the defect:

(9) $$E_{t}=-q\frac{d}{t_{\mathrm{ox}}}V_{G}+C.$$

At the gate voltage $V_{G,i}$, where the measured capture and emission times intersect, the trap level is equal to the channel Fermi level at this voltage $E_{t}=E_{f,i}$.:

(10) $$E_{f,i}=-q\frac{d}{t_{\mathrm{ox}}}\left(V_{G,i}\right)+C.$$

Finally, substituting *C* from Equation (10) in Equation (9) and evaluating at $V_{G}=-V_{\mathrm{FB}}-V_{S}$ yields the trap level at zero field

(11) $$E_{t},0=E_{f,i}+q\frac{d}{t_{\mathrm{ox}}}\left(V_{G,i}-\phi_{s}-V_{\mathrm{FB}}\right),$$

with the oxide electric field $F_{\mathrm{ox}}$, the surface potential $\phi_{s}$ and flatband voltage $V_{\mathrm{FB}}$.

## 3. Results

Based on the methodology presented above, the recorded RTN traces are analyzed. The extracted noise parameters are used to find the distributions of step height, vertical defect position and energy level.

### 3.1. Threshold Voltage Shift and Number of Defects

**Figure 5:**
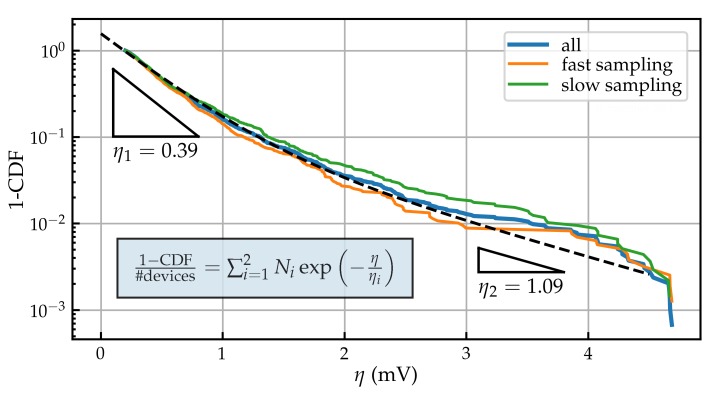
Complementary cumulative density function (1-CDF) of the step heights η extracted from our measurements, shown for both the slow and the fast sampled data. The distribution seems to be bimodal, composed of a defect distribution with a smaller average impact $\eta_{1}$ and a distribution with a larger impact $\eta_{2}$.

A plot showing the complimentary cumulative density function (1-CDF, or CCDF) of the step heights extracted from the measurement data of approximately 300 devices is shown in Figure 5. For a single defect distribution, an exponential distribution of step heights, i.e., a straight line in the CCDF plot, is expected [28]. In this case, however, the measurements seems to comprise two separate distributions of defects. As a consequence, defects with responses between 3 mV to 5 mV seem slightly more common than expected.

### 3.2. Energy and Position

**Figure 6:**
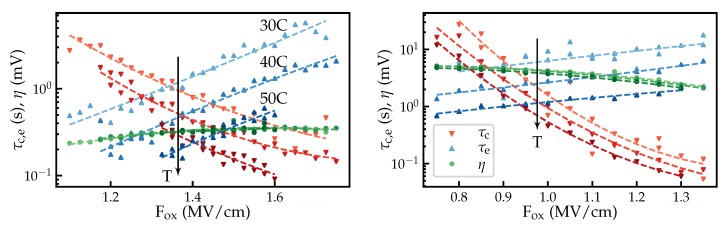
Two defects characterized in detail. From the intersection points and the steepness of the charge capture and emission times, the positions and the trap levels of the defects can be estimated.

Examples of defects characterized in more detail are shown in Figure 6. Using the method described in Section 2.3, trap levels and positions of defects are estimated for around 100 defects.

**Figure 7:**
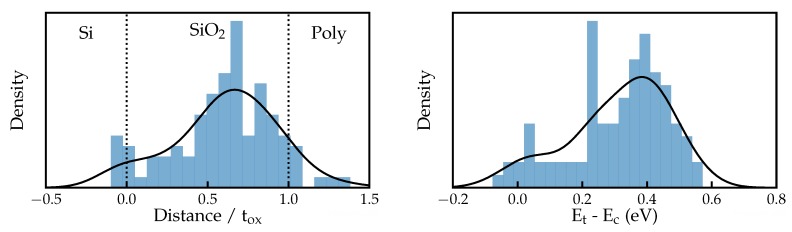
Distributions of positions and trap levels extracted using Equation (8) and Equation (11). Note that the measured distribution in position should not be interpreted as the complete distribution of defects in the device. It is rather a result of the energetic distribution of the defects in conjunction with the characterization window, which is diagonal in energy and distance, given by the Fermi level at measurement conditions.

The results are shown in histograms for distance and trap level in Figure 7. Most of the defects which have been characterized are found between 0.5 $t_{\mathrm{ox}}$ and 0.8 $t_{\mathrm{ox}}$, which seems to be the depth at which the electron defect band corresponds with the Fermi level at the measurement bias. This places the trap level of the extracted defects around 0.4 eV above the Fermi level, which according to our numerical device simulations is close to the Si conduction band.

As can further be seen in Figure 7, the estimation given by Equation (8) suggests some defects to be located outside the oxide. This is due to a number of shortcomings in this methodology:

(i) The estimation only accounts for interaction with the channel, defects interacting primarily with the gate might have inverted capture and emission time behavior which results in a negative distance.
(ii) The prefactor which is assumed as constant in the estimation does change with the logarithm of the channel carrier density. This leads to some overestimation of the distance.
(iii) The estimation is based on the values and first derivatives of the capture and emission times at the intersection point. Measurements which do not show $\tau_{c}=\tau_{e}$ within the measurement window need to be extrapolated, which leads to inaccuracies.
(iv) Some defects may not be adequately described using a two state model [29].

**Figure 8:**
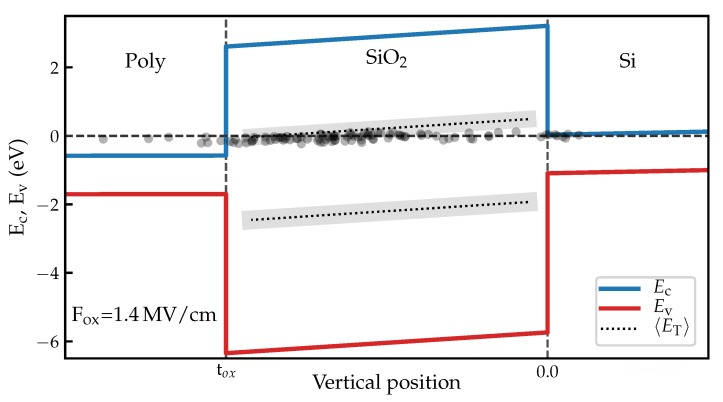
Simulated band diagram showing the locations of defects extracted using Equation (8) and Equation (11). In addition, defect bands as given in [30] are shown in comparison. Notice how some defects appear located outside of the SiO${}_{2}$ layer. This shows the shortcomings of the estimation used. The estimation does not account for defect interaction with the gate, which often results in negative $d/t_{\mathrm{ox}}$ and it generally overestimates the distance of the defects due to the neglected prefactor.

In Figure 8, the extracted defects are shown in a simulated band diagram. Furthermore, defect bands which were obtained from positive bias temperature instability (PBTI) and negative bias temperature instability (NBTI) experiments on n- and pMOS transistors, respectively, are indicated at energy values taken from [30].

For the electron trap band this is around 0.5 eV above the conduction band, which is around 0.1 eV higher than the values we extract from the measured defects. This might be due to defects closer to the gate interacting primarily with the gate instead of the channel, effectively limiting our measurement range in energy. A number of defects show up with distances around zero, i.e., with very small bias dependencies of their $\tau_{c}/\tau_{e}$ ratios. They may be located close to either the Si/SiO${}_{2}$ interface or to the SiO${}_{2}$/poly interface.

### 3.3. Simulation

**Figure 9:**
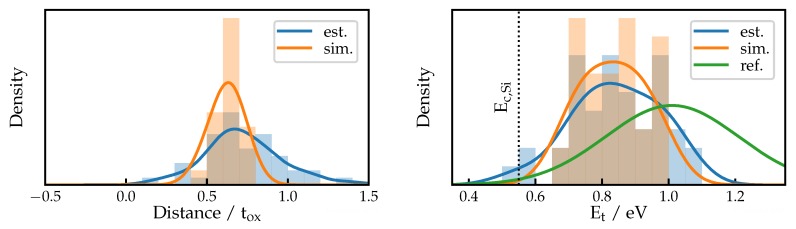
Distributions of positions and trap levels from technology computer aided design (TCAD) simulations compared to the estimations made for the same defects. Energies are referenced from Si-midgap, with $E_{f},i$ taken from the simulation. The electron defect band at $E_{t}=1.01\;eV$ from [30] is shown for comparison. The defects we observe are distributed mainly in the lower half of this band.

To obtain more accurate data, the capture and emission time behavior for a subset of the defects characterized using the estimation formulas is replicated using technology computer aided design (TCAD) simulation [31]. For the simulations, an effective two-state nonradiative multi phonon (NMP) model is used. Only those measurements are used where the intersection point lies within the measurement range. This removes the peak close to the interface observed in Figure 7 and Figure 8. The distributions for the resulting distances and energies are shown in Figure 9.

It can be seen that the simulation results are in good agreement with the data obtained from the estimations. The average depth assigned to the measured defects is slightly lower in the simulation and all defects are confined to the oxide. It can be further observed that the average of the energy distribution is slightly lower compared to the estimation. Compared to the electron defect band at $E_{t}=1.01\;eV\pm 0.218\;eV$ from [30], which was obtained from PBTI experiments on nMOS transistors, we extract only defects in the lower half of this band, as only those can contribute to the measured RTN.

## 4. Discussion

The distributions of step heights in Si/SiO${}_{2}$ devices which have been reported in literature can commonly be explained using a single exponential distribution. However, in the underlying work we clearly observe a bimodal distribution of the measured step heights, which one would not expect for Si/SiO${}_{2}$ devices. Bimodal distributions are commonly observed for PBTI only in high-k devices, where they are thought to originate from traps in both the bulk high-k layer and the interstitial layer [32,33,34]. A simple approximation of the threshold voltage shift expected is possible using the charge sheet approximation, which gives values below 0.2 mV for these devices. This agrees within the limits of this approximation to the smaller $\eta_{1}$ observed. However, the origin of the larger $\eta_{2}$ in this technology needs further investigation.

In this work, we use an analytical approximation to extract the trap levels and spatial distribution from a large set of RTN measurements and compare the results to TCAD simulations. The results for the energetic and spatial distributions show good agreement between the estimated and the simulated parameters, considering the approximations made in the estimation. The energetic distribution of the extracted defects covers the lower half of an electron defect band obtained from simulations reported recently. Due to the limited scanning range of the RTN measurements performed, no defects could be measured in the upper half of this band. A possibility to extend the energetic range of the investigations in a future work could be the combination of the obtained RTN data with data obtained from TDDS measurements.
